# Genome-wide terpene gene clusters analysis in Euphorbiaceae

**DOI:** 10.1093/hr/uhaf097

**Published:** 2025-05-23

**Authors:** Yinhang Wang, Yunxiao Zhao, Ming Gao, Yangdong Wang, Wei Li, Yicun Chen

**Affiliations:** State Key Laboratory of Tree Genetics and Breeding, Chinese Academy of Forestry, Beijing 100091, China; Research Institute of Subtropical Forestry, Chinese Academy of Forestry, Hangzhou, Zhejiang 311400, China; State Key Laboratory of Tree Genetics and Breeding, Northeast Forestry University, Harbin 150040, China; State Key Laboratory of Tree Genetics and Breeding, Chinese Academy of Forestry, Beijing 100091, China; Research Institute of Subtropical Forestry, Chinese Academy of Forestry, Hangzhou, Zhejiang 311400, China; State Key Laboratory of Tree Genetics and Breeding, Chinese Academy of Forestry, Beijing 100091, China; Research Institute of Subtropical Forestry, Chinese Academy of Forestry, Hangzhou, Zhejiang 311400, China; State Key Laboratory of Tree Genetics and Breeding, Chinese Academy of Forestry, Beijing 100091, China; Research Institute of Subtropical Forestry, Chinese Academy of Forestry, Hangzhou, Zhejiang 311400, China; State Key Laboratory of Tree Genetics and Breeding, Chinese Academy of Forestry, Beijing 100091, China; State Key Laboratory of Tree Genetics and Breeding, Northeast Forestry University, Harbin 150040, China; State Key Laboratory of Tree Genetics and Breeding, Chinese Academy of Forestry, Beijing 100091, China; Research Institute of Subtropical Forestry, Chinese Academy of Forestry, Hangzhou, Zhejiang 311400, China

## Abstract

Euphorbiaceae species are renowned not only for horticultural significance but for their production of numerous bicyclic diterpenes with antitumor and antiviral activities. However, the gene clusters responsible for the biosynthesis of these terpenes remain largely unidentified. We here initiated the construction of a comprehensive procedure for terpene gene clusters in Euphorbiaceae species. A total of 1824 candidate gene clusters with the range of 30–800 kb were identified across seven representative species including *Ricinus communis*, *Hevea brasiliensis*, *Euphorbia peplus*, *Jatropha curcas*, *Manihot esculenta*, *Vernicia montana*, and *Vernicia fordii* in Euphorbiaceae. The 16 high-confidence terpene gene clusters were ultimately pinpointed in Euphorbiaceae after satisfied the three stringent screening criteria: TPS/CYP pairwise relationship, copathway and coexpression patterns. Notably, the well-known casbene and casbene-derived diterpenoid gene cluster, involved in the biosynthesis of casbene, neocembrene, ingenanes, and jatrophanes, were identified. It was observed that casbene gene clusters were universally presented in Euphorbiaceae species, except *M. esculenta*. Among the casbene gene cluster, the alcohol dehydrogenase (ADH) was initially appeared, and neocembrene synthase is exclusively present in *R. communis* while absent in all the other species. These findings represent a significant step toward understanding the genetic basis of terpene biosynthesis in Euphorbiaceae species. Moreover, this knowledge on gene clusters responsible for the biosynthesis of pharmacologically relevant terpenes can serve as a theoretical foundation for future applications.

## Introduction

Euphorbiaceae is a widely distributed plant family with ~300 genera and 5000 species, mainly in tropical and subtropical regions. Plants of this family include trees, shrubs, and herbs, generally having milky sap, usually alternate leaves, unisexual flowers, commonly monoecious or dioecious, and fruit mostly capsules. As a horticultural ornamental plant, the wide variety of plants in the Euphorbiaceae family, from low-growing herbs to tall shrubs and succulents, can show their unique charm in different horticultural scenes. Euphorbiaceae not only shine in horticulture but also hold immense promise for medicinal applications and biochemical resources. It is well known for its diverse array of chemical components, including terpenoids, flavonoids, coumarins, steroids, polyphenols, and other compounds. Particularly, notable diterpenoids have been identified from this family. So far, >650 macrocyclic diterpenoids from 21 skeleton types have been isolated from Euphorbiaceae species. It mainly includes cembrabe/casbene, jatrophanes, ingenanes, lathyranes, myrsinols, abietane, tiglianes, berulane, daphnane, atisanetype, and ent-kaurane, etc [1]. These compounds are increasingly recognized for their potential in combating multidrug resistance and their effectiveness in tumor chemotherapy [2]. For example, ingenol mebutate, derived from *Euphorbia peplus*, has been used to treat actinic keratosis and has shown promise in clinical trials for treating superficial basal cell carcinoma [3]. Harnessing big data methodologies to establish a robust gene cluster prediction framework for Euphorbiaceae species could significantly advance research and applications in this area.

Similar to prokaryotic manipulators, clusters of functionally related, nonhomologous genes also occur in the genomes of eukaryotes, such as yeast, fungi, and insects. These gene clusters are involved in a variety of secondary metabolic pathways. In recent years, there has been increasing identification of gene clusters responsible for synthesizing these secondary metabolites in some model plant species [4]. The rapid advancement of genomic, transcriptomic, and metabolomic technologies has empowered researchers to utilize algorithms and software programs for high-throughput mining of metabolic gene clusters (Chen *et al.*, 2013; [5]). Based on a wealth of published plant omics data, systematic algorithms have been developed to efficiently identify potential metabolic gene clusters in plants [4,5]. Schläpfer *et al.* [6] developed PlantClusterFinder, a novel prediction software, to analyze gene clusters across 18 plant species. Similarly, algorithms such as PlantiSMASH [7] and PhytoClust [8] utilize profile Hidden Markov Models (pHMMs) to accurately identify biosynthetic enzymes and predict gene clusters based on genomic data. Integrating gene coexpression data into these models has been shown to refine predictions by reducing the number of candidate gene clusters while significantly improving accuracy [9]. In summary, ongoing advancements in predictive capabilities for identifying gene clusters, coupled with the continuous refinement of gene cluster features and computer algorithms, are set to further enhance our understanding.

To identify terpene gene clusters responsible for the biosynthesis of macrocyclic diterpenoids in Euphorbiaceae plants is highly significant. This work not only enriches our understanding of the biosynthesis of Euphorbiaceae terpene, but also provides a new research direction for the subsequent metabolic engineering transformation. Here, we have developed a comprehensive gene cluster prediction framework tailored for Euphorbiaceae, with a specific focus on terpene gene clusters. This framework integrates genomic, transcriptomic, and metabolic pathway databases to enhance our understanding. Our research delved into the evolutionary analysis of casbene gene clusters, and conducted a detailed examination of the tissue-specific distribution of casbene gene clusters. This approach not only enriches our knowledge of terpene biosynthesis pathways in Euphorbiaceae but also offers valuable tools for future investigations and applications in pharmacology and agriculture.

## Results

### An effective genome-wide gene cluster prediction process in Euphorbiaceae

**Figure 1:**
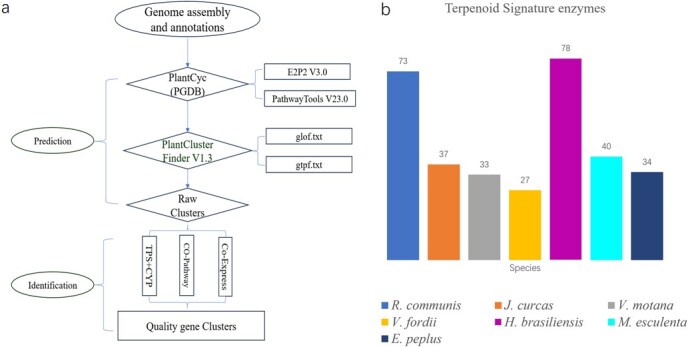
The process of genome-wide terpene gene cluster prediction and the number information of predicted TPSs in Euphorbiaceae. (a) The process of terpene gene cluster prediction and identification. PlantCyc (PGDB) metabolic pathway database was constructed using E2P2 enzyme prediction and PathwayTools metabolic pathway. For identification, the gff annotation file was preprocessed to obtain glof.txt and gtpf.txt files, which was combined with PGDB to predict a large number of raw gene clusters using PlantClusterFinder. The identification steps included coexpression and copathway screening, and finally high-quality gene clusters were obtained. (b) The number of TPS enzymes. The information of TPS enzymes were obtained through the prediction process.

The integration of advanced tools like E2P2 v3.0, Pathway Tools, and PlantClusterFinder has significantly strengthened our gene cluster prediction framework for Euphorbiaceae species (Fig. 1a). By leveraging these tools, we were able to predict a substantial number of candidate gene clusters—1824 in total—spanning across 187 804 genes from seven representative species: *Ricinus communis*, *Hevea brasiliensis*, *E. peplus*, *Jatropha curcas*, *Manihot esculenta*, *Vernicia montana*, and *Vernicia fordii* (Table 1).

**Table 1:** Genome-wide terpene gene cluster prediction results in Euphorbiaceae species.

Species	Genomic size (Mb)	Cluster number	Gene number
*Ricinus communis*	305	254	20 512
*Jatropha curcas*	258	262	19 104
*Vernicia montana*	1200	213	27 465
*Vernicia fordii*	1200	260	28 945
*Hevea brasiliensis*	1800	386	36 622
*Manihot esculenta*	619	332	29 682
*Euphorbia peplus*	260	117	25 474
Total	5642	1824	187 804

**Table 2:** Metabolic pathway database statistics on Euphorbiaceae species.

Species	Enzyme	Enzymatic	Pathways	Compounds
*R. communis*	8024	2704	438	2224
*J. curcas*	7156	2633	421	2150
*V. motana*	5868	2680	424	2198
*V. fordii*	5963	2761	442	2268
*H. brasiliensis*	12 698	2754	440	2252
*M. esculenta*	11 176	2722	434	2212
*E. peplus*	6395	2689	425	2225
Total	57 280	18 943	3024	15 529

The statistical analysis of our prediction results reveals that the physical lengths of the predicted candidate gene clusters for Euphorbiaceae species fall within the range of 30–800 kb. Specifically, we observed an intermediate value of 148 kb and an average length of 184 kb across these clusters. Furthermore, the number of gene clusters predicted per species ranges from 8 to 31. These findings are consistent with previous research reports on plant secondary metabolism gene clusters, as documented by Field and Osbourn (Field and Osbourn *et al.*, 2008) and Frey [10]. These studies have shown that such gene clusters typically exhibit physical lengths ranging from 30 kb to several hundred kilobases, reflecting the complexity and diversity of biosynthetic pathways involved in producing secondary metabolites.

By aligning our results with established literature, we validate the robustness of our prediction framework and underscore its relevance in advancing our understanding of gene cluster organization and evolution within the Euphorbiaceae family. This statistical clarity not only enhances our predictive models but also provides a solid basis for further exploration into the functional characterization and exploitation of these gene clusters for biotechnological applications.

The predicted physical lengths of the 1824 gene clusters ranged widely from 1 to 1337 kb, with a median length of 90 kb, and 89% of the clusters were <300 kb (Fig. S1). For experimentally verified plant metabolite synthesis gene clusters, typical lengths fall between 33 and 284 kb, such as the triterpenoid thalianil synthesis gene cluster in *Arabidopsis thaliana* (33 kb) and the benzoxazolizinone synthesis genes in maize (264 kb) [10,11]. In Euphorbiaceae species, 66.7% (1217) of identified clusters also fell within this range. The number of genes within all clusters ranged from 3 to 54, with a median of 9 genes. Among these, 85% (1552 clusters) contained 3–18 genes. Known plant metabolic gene clusters typically contain between 4 and 18 genes [6].

### Identification of high-confidence terpene gene clusters in Euphorbiaceae

Out of the initial 1824 predicted gene clusters, those with high confidence in terpene synthesis underwent further screening (Fig. S1), focusing on the presence of ‘signaling’ enzymes within the Euphorbiaceae family. Pathway Tools integrated with PGDB (Pathway/Genome Databases) in PlantClusterFinder facilitated the annotation of various ‘signaling’ enzymes, including terpenoids (TPS), phenylpropanoids (PSE), alkaloids [12], and polyketides (PKS) within these gene clusters (Fig. S2). Metabolic pathway databases specific to each species were constructed using E2P2 and Pathway Tools software (Table 2). *Hevea brasiliensis* showed the highest diversity with 3024 pathways and 15 529 compounds, whereas Vernicia species exhibited comparatively fewer pathways. Generally, there was a positive correlation observed among enzymes, pathways, and compounds.

As part of the study objectives, a total of 322 terpene signaling enzymes were initially identified across seven species of the Euphorbiaceae family (Fig. 1b). Secondary metabolite synthesis gene clusters encompass genes encoding enzymes involved not only in synthesizing the backbone of secondary metabolites (referred to as ‘signal’ enzymes) but also in modifying intermediates (‘tailoring’ genes). These ‘signal’ genes organize into metabolic clusters by recruiting ‘tailoring’ genes around them, which synergistically catalyze the production of final products [13].

In conjunction with a rigorous gene cluster screening approach, a total of 16 high-confidence terpene gene clusters were ultimately identified in Euphorbiaceae species. This selection process involved applying three stringent screening criteria: TPS/CYP pairwise relationship, copathway analysis, and coexpression patterns (Table 3).

**Table 3:** Comparison on the gene cluster numbers before and after screening.

Species	Raw cluster	TPS + CYP	Copathway	Coexpress	Quality cluster
*R. communis*	254	10	8	6	1
*J. curcas*	262	8	3	3	1
*V. motana*	213	14	9	5	2
*V. fordii*	260	7	5	3	2
*H. brasiliensis*	386	16	12	12	1
*M. esculenta*	332	7	5	3	1
*E. peplus*	117	11	11	8	8
Total	1824	72	52	38	16

**Figure 2:**
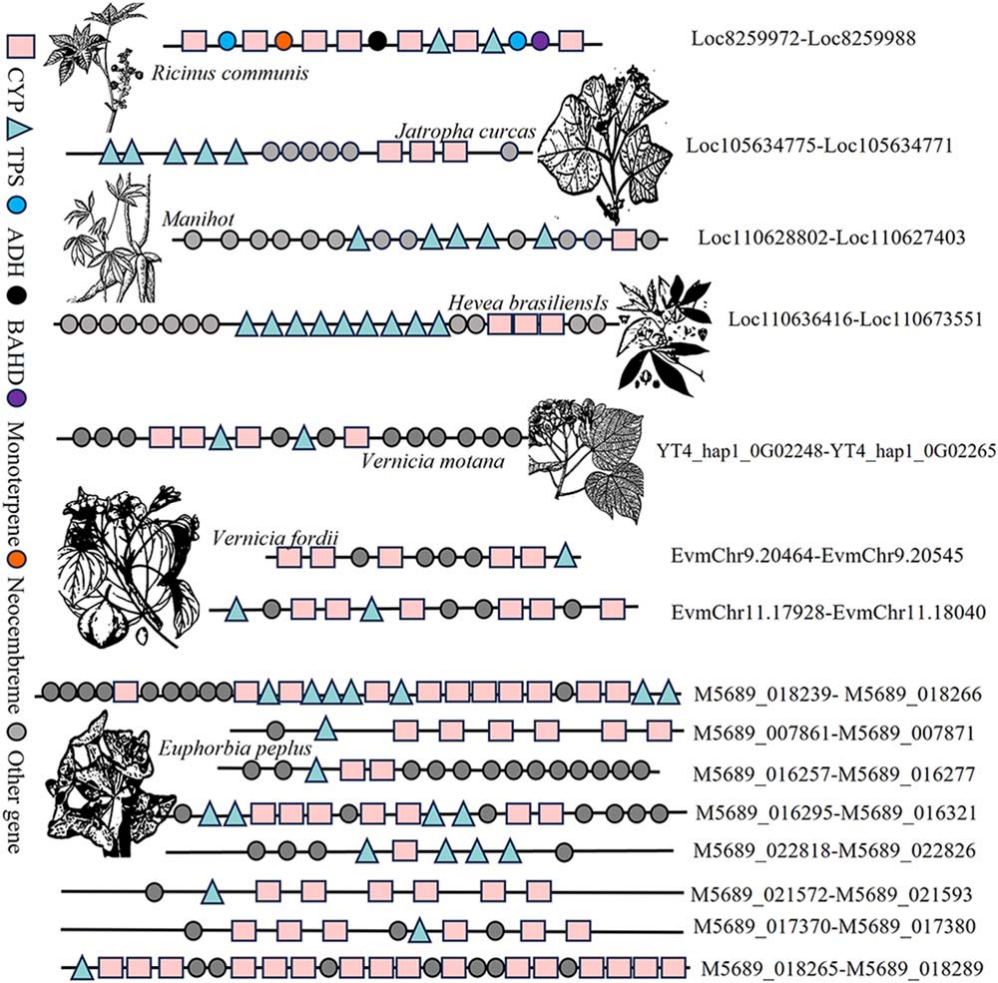
Graphic results of 16 predicted high-quality terpene gene clusters in Euphorbiaceae species. Eight high-quality gene clusters were identified in *E. peplus*. And two high-quality gene clusters in *V. fordii*. One high-quality gene cluster was identified in all other Euphorbiaceae species. The identified *R. communis* gene cluster (Loc8259972-Loc8259988) and *E. peplus* gene cluster (M5689_018265-M5689_018289) have been reported as casbene gene clusters.

Among the 16 high-confidence terpene gene clusters (Fig. 2), the number of genes within these clusters ranged from 8 to 30. In *R. communis*, the casbene gene cluster (Loc8259972-Loc8259988) was identified on Chromosome 4 and is involved in the synthesis pathway of ricinoleic acid. In *J. curcas*, specific TPS genes were found in predicted gene clusters contributing to the synthesis pathways of lupulinol (5.4.99.41) and β-carvacryl alcohol (5.4.99.39). *Hevea brasiliensis* gene clusters contained TPS genes participating in two downstream branch pathways of casbene (4.2.3.15, 4.2.3.111). In *V. montana*, two predicted gene clusters included genes involved in the synthesis of monoterpene products such as nerolidol and rosinol. Eight highly confident gene clusters were identified in *E. peplus*, including the leprosy alkane cluster (M5689_018265-M5689_018289), consistent with previous reports [14].

### Analysis of casbene and casbene-derived diterpenoid gene clusters in *R. communis* and *E. peplus*

Through genome-wide prediction in *R. communis*, a total of 253 gene clusters were initially identified. Among these, 10 clusters were selected based on the presence of both ‘signaling’ and ‘trimming’ enzymes involved in terpene biosynthesis. Further scrutiny revealed that six out of these 10 clusters satisfied both copathway and coexpression conditions, indicating their likely involvement in specific terpene synthesis pathways within the plant.

**Figure 3:**
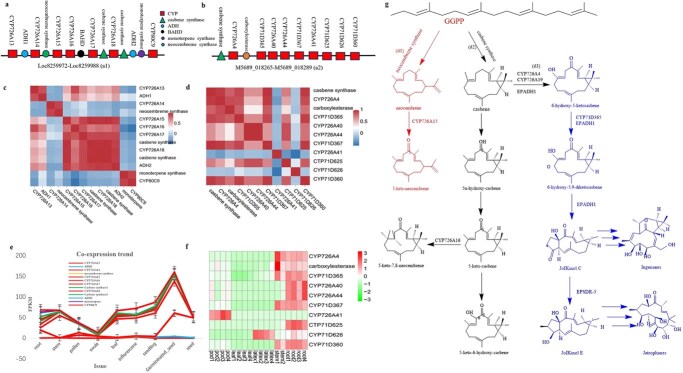
The casbene and casbene-derived diterpenoid gene cluster in *R. communis* and *E. peplus*. (a) Casbene gene clusters in *R. communis.* (b) Casbene-derived diterpenoid gene cluster in *E. peplus*. (b) Pearson coexpression trend heat map on casbene cluster in *R. communis* (c) and casbene-derived diterpenoid gene cluster in *E. peplus* (d). (e) Casbene gene cluster were highly coexpressed in seedling of *R. communis*. Root, 2 weeks after pollination; stem, 2 weeks after germination; pollen, 3 days after development; ovule, 3 days after development; leaf, 2 weeks after germination; inflorescence, 5 days after tissue formation; seedling, 2 weeks after germination; germinated seed, 5 days after germination; seed, 20 days after pollination. (f) *Euphorbia peplus* CYP726A626 is highly expressed in latex tissue. (g) Pathway for the biosynthesis of casbene, neocembrene, ingenanes, and jatrophanes.

The casbene gene cluster on Chromosome 4 of *R. communis* is a notable cluster encompassing 13 genes, each playing a crucial role in terpene biosynthesis. This cluster (Fig. 3a) includes two ‘Signaling’ enzymes, specifically the diterpene casbene synthases (LOC8259981 and LOC8259984), which initiate the synthesis pathway. Additionally, there are six P450 ‘trimming’ enzymes within the cluster: LOC8259988 (CYP80C9), LOC8259983 (CYP726A18), LOC8259978 (CYP726A16), LOC8259976 (CYP726A15), LOC8259974 (CYP726A14), and LOC8259972 (CYP726A13). The cluster also contains two alcohol dehydrogenase (ADH) genes (LOC8259973 and LOC8259985) involved in alcohol metabolism, a BAHD acyltransferase likely responsible for acyl group transfers, a monoterpene synthase (LOC8259987), and a sesquiterpene synthase (LOC8259975), contributing to the diversity of terpene products synthesized.

Analysis of transcriptome data across various tissues—roots, stems, leaves, and seeds—revealed differential expression patterns. A Pearson correlation test highlighted strong coexpression relationships between the P450 genes and the casbene synthases (Fig. 3c), indicating coordinated regulation of these genes within the pathway. The expression of the casbene gene cluster was notably higher in leaves and seeds compared to other tissues (Fig. 3e). Furthermore, overall, the casbene gene cluster in *R. communis* represents a complex network of genes involved in terpene biosynthesis, with distinct expression patterns across different tissues, emphasizing its biological significance in the production of specialized metabolites such as ricinoleic acid.

The oxidation of casbene-5 is a critical and highly conserved step in the biosynthesis of bioactive diterpenes within the Euphorbiaceae family. Various CYP726A genes, such as CYP726A14, CYP726A17, and CYP726A18 from *R. communis*, play a crucial role in catalyzing this process. They are responsible for converting casbene into 5-keto-Casbene and 5α-hydroxy-Casbene [15]. Additionally, CYP726A16 has been identified to catalyze the 7,8-epoxidation of 5-keto-casbene. Meanwhile, CYP726A19 from *E. peplus* serves as a casbene 5-oxidase, yielding similar products to those of CYP726A14 but with differing proportions of hydroxy- and keto-products (Zhang *et al.*, 2023). These oxidation processes are not limited to casbene alone but are observed across various biologically active diterpenes produced by Euphorbiaceae plants. The modification of position five appears to be universally conserved and crucial for the chemical diversity and biological activities of these compounds [14]. Moreover, CYP726A14 exhibits remarkable versatility by not only performing the initial hydroxylation but also catalyzing subsequent reactions leading to the formation of 5-keto-carbenes and 5-keto-6-hydroxy-carbenes from 5-hydroxy-carbenes [16]. The gene cluster involved in the pathways of diterpene biosynthesis in Euphorbiaceae were illustrated as Fig. 3g.

Interestingly, within the same gene cluster, CYP726A15 demonstrates the ability to oxidize the C-5 position of neocembrene in two consecutive steps, resulting in the production of 5-keto-neocembrene [15]. These findings underscore the intricate enzymatic machinery involved in diterpene biosynthesis, highlighting the specificity and diversity of CYP726A enzymes in modifying terpene structures to generate pharmacologically relevant compounds.

We identified another gene cluster responsible for casbene-derived diterpenoids in *E. peplus* (Fig. 3b). Using coexpression and copathway screening criteria, complemented by Pearson correlation analyses, we found compelling evidence supporting the existence and functionality of this gene cluster (Fig. 3d). Transcriptome analyses revealed that several cluster genes, such as CYP726A4 and CYP71D365, exhibit peak expression levels in stems and pods [14]. Conversely, CYP71D626 demonstrated predominant expression in latex (Fig. 3f). Furthermore, studies on *E. peplus* have employed techniques like viral-induced gene silencing to elucidate the biosynthetic pathways of jatrophane and ingenane diterpenes (Fig. 3g), contributing to a clearer model of biosynthesis within the Euphorbiaceae family [14].

### The evolution analysis of casbene gene clusters in Euphorbiaceae

**Figure 4:**
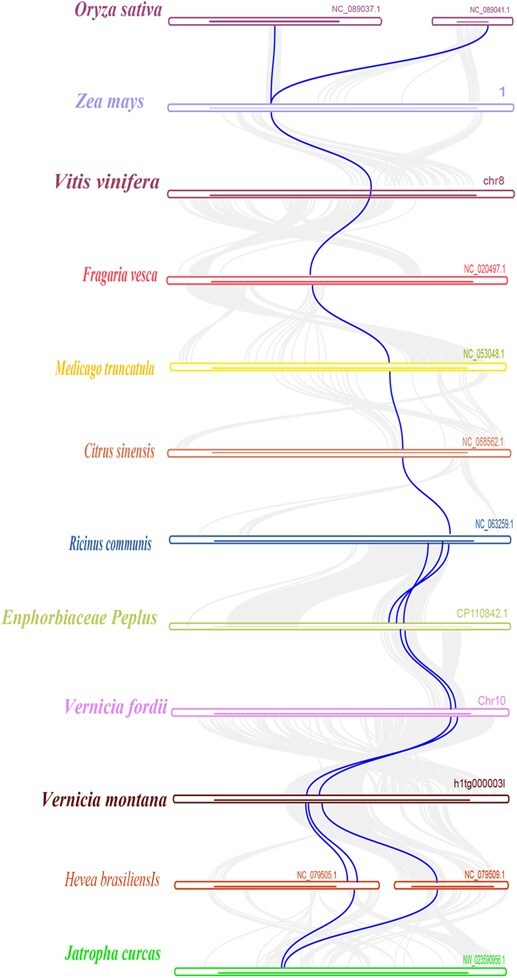
Collinearity analysis of casbene gene clusters in Euphorbiaceae species. The ancestral collinearity. relationships within casbene gene clusters are indicated by bold connections, while other syntenic gene relationships are shown with secondary connections. The analysis reveals stronger retention of genes within casbene clusters across multiple Euphorbiaceae species, including *R. communis, E. peplus, V. fordii, V. montana, H. brasiliensis, and J. curcas..*

To investigate the evolution of casbene gene clusters in angiosperm, we analyzed species harboring these clusters using the phylogenetic tree based on the APG IV classification of flowering plants. The results of collinearity showed that the complete castor gene cluster specifically appeared in Euphorbiaceae plants (Fig. 4).

The collinearity analysis of casbene gene clusters (Fig. 4) indicated that most species in Euphorbiaceae have more or less collinearity, except in cassava (*M. esculenta*). The result was consistent with the previous study [12,17]. *Manihot esculenta*, assigned to the Crotonoideae, is a staple crop in the diet of an ~800 million people in Africa, Asia, and the Americas [18]. The bioactive diterpene products derived from the same casbene or neocembrene have not been reported yet in *M. esculenta*. A number of molecular phylogenetic studies have shown that *M. esculenta* does not form a single monophyletic group with the other Crotonoideae [12,17]. Therefore, this genus may have evolved independently from other diterpene-producing Crotonoideae, or it may have initially co-evolved and subsequently lost the diterpene biosynthesis gene cluster [15]. Meanwhile, the results also indicated that more collinear genes of the casbene cluster were retained in *Vernicia* plants within Euphorbiaceae (such as *V. montana* and *V. fordii)*.

In our multispecies collinearity analysis, the *ADH* gene showed significant collinearity across all species (Fig. 4). This means that the location and structure of the ADH gene is highly consistent across species. This collinearity suggests that the ADH gene may be highly conserved and important in these species, making it a unique target for analysis in our study. ADHs, belonging to the medium-chain oxygenase/reductase family, could catalyze reciprocal transformations between alcohols and aldehydes [19]. For example, ADHs have been shown to catalyze citral production from geraniol and nerol [20]. The potential role of ADH genes involved in Euphorbiaceae terpene metabolism is well worth exploring.

**Figure 5:**
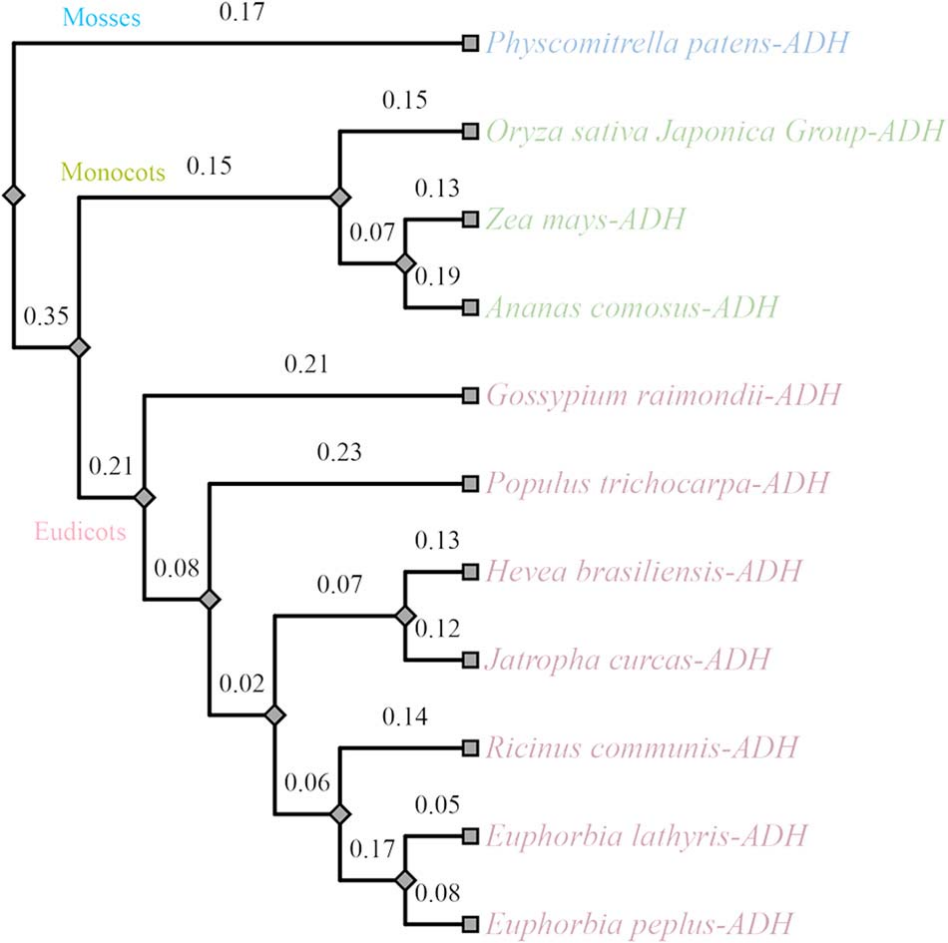
Evolution of ADH gene among angiosperms. The ADH gene in the castor gene cluster was originated from the early mosses, and continuously possessed by monocots and eudicots species.

The ADH gene in castor gene cluster was originated from the earliest in angiosperms (Fig. 4), and continuously possessed by mosses, monocots, and eudicots species (Fig. 5). A significant number of collinear genes, particularly casbene synthase and P450 genes, are predominantly appeared within Euphorbiaceae species. The genes meet the criteria for forming gene clusters in certain species, as exemplified by the casbene gene clusters observed in *E. peplus* [14], consistent with previous reports highlighting their prevalence in Euphorbiaceae [15].

### Casbene gene cluster exclusively expressed in roots of *Vernicia*

**Figure 6:**
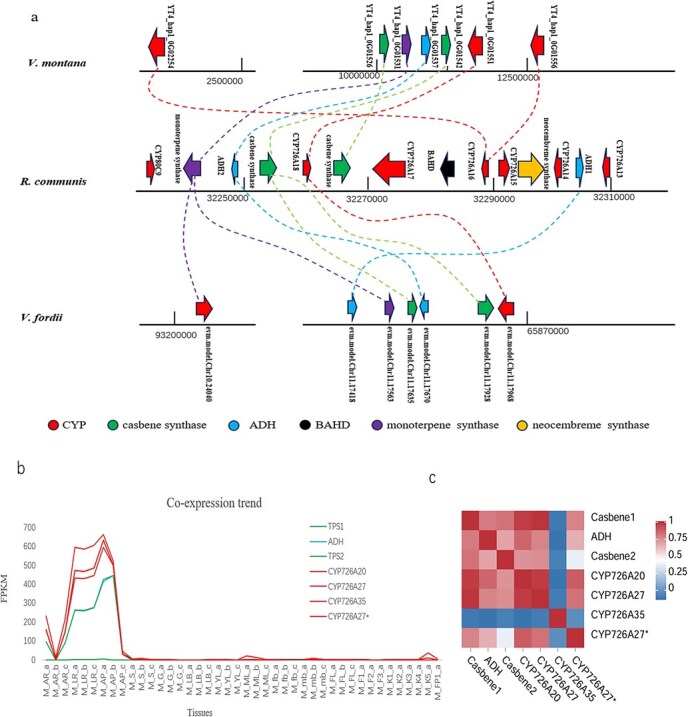
Analysis of casbene gene clusters in *Vernicia*. (a) The structure of casbene gene clusters in *Vernicia*. (b) Casbene gene clusters synergistically coexpressed in the root of *V. montana*. (c) Pearson correlation heat map. According to the multitissue coexpression thermogram, tissues in the root showed a high degree of coexpression in *V. montana*. AR, main root; LR, lateral root; AP, root tip; S, stem; G, glandular body; LB, lateral branch; YL, young leaves; ML, mature leaves; fb, female bud; mb, male bud; FL, small fruit; F, fruit; K, seed kernel; FP1, fruit peel.

The casbene gene clusters of *V. montana* and *V. fordii* showed significant collinearity compared to other species within Euphorbiaceae (Figs 4 and 6a). Motivated by this observation, we investigated the presence of casbene gene clusters in *V. fordii* and *V. montana*.

*Vernicia fordii*, a long-cultivated oilseed tree known for its high-quality tung oil, has been extensively used in industry, agriculture, fishery, military, and medicine. We conducted correlation analyses of the terpene gene clusters in *V. fordii*, which are illustrated (Fig. S3). The results showed that the collinearity homologous genes of *V. fordii* were not coexpressed in the clusters, so we determined that there were no terpenoids forming gene clusters.

*Vernicia montana* is renowned for its robust disease resistance and other distinctive characteristics. Therefore, our study centered on analyzing the terpene gene clusters of *V. montana* (Fig. 6a). *Vernicia montana* clusters consist of two casbene synthases (YT4_hap1_0G01526, YT4_hap1_0G01542), one monoterpene synthase (YT4_hap1_0G01537), and three P450 genes CYP726A20 (YT4_hap1_0G01551), CYP726A35(YT4_hap1_0G01556), and CYP726A27 (YT4_hap1_0G02254).

In particular, the casbene produced were generally found in *R. communis* seedlings (Sitton *et al.*, 1975) and *E. peplus* seeds [15]. However, this is not the case in *Vernicia*. Analysis of multiple transcriptome datasets across various tissues indicated that the gene cluster is exclusively expressed in root tissues in *V. montana* (Fig. 6b). Furthermore, a Pearson correlation test highlighted significant coexpression between the casbene synthase (TPS) and the P450 genes (CYP) (Fig. 6c).

Moreover, in terms of the gene cluster structure, the neocembreme synthase was missing in casbene gene cluster of *Vernicia*. Correspondingly, CYP726A15, which catalyzes the oxidation of neocembreme to produce 5-Keto-neocembreme (Schlapfer *et al.*, 2017), is also missing. Therefore, the branch pathway (Fig. 3g) involved in biosynthesis of neocembreme does not exist in *Vernicia*. This change indicated that *Vernicia* may rely on other alternative metabolic pathways.

## Discussion

### Debugging of genome-wide gene cluster prediction error reporting in Euphorbiaceae

The software's operational results can vary significantly based on different parameter configurations, necessitating careful consideration and problem analysis during execution. This discussion primarily focuses on several common and effective methods for adjusting parameters when encountering errors.

#### Software environment

PlantClusterFinder is a powerful tool for genome-wide gene cluster prediction, but it comes with high computational costs and requires a Linux environment, specifically Ubuntu version 22.04. Originally based on MATLAB, the software has evolved significantly since its early versions. Starting from the 2018 update (version 1.3), the requirement for MATLAB has shifted to the latest version, MATLAB-2023b, due to enhanced functionalities and compatibility needs. Additionally, the package now recommends PlantClusterFinder-python_gap.

#### Input file format error

In the input files glof.txt and gtpf.txt, each column of data must be separated by a single tab. In genomic and annotation datasets, some genes may lack corresponding protein IDs, resulting in discrepancies in gene counts between glof.txt and gtpf.txt files. However, software operations demand that the number of genes in both files match. Therefore, researchers must ensure beforehand that the gene counts in glof.txt and gtpf.txt are synchronized to maintain consistency.

#### Output PGDB error

If researchers encounter issues where the output PGDB file from Pathway Tools appears blank, it could be due to the metabolic pathway database not being properly linked to the corresponding data. To resolve this, it is recommended to select the metabolic pathway database specific to the species of interest through the Home interface. Navigate to Files → Import → DB Links from file, and import the corresponding data files (ending with .dat). After importing, attempt to reexport the PGDB file. If errors persist during software operation despite following these steps strictly, one potential solution is to modify the Protein Name prefixes in the gtpf.txt file. For instance, changing ‘XM_012064637.1’ to ‘012064637.1’. Ensure that corresponding adjustments are made in your PGDB to reflect these changes. These recommendations stem from insights gained through multiple communications with software developers while troubleshooting similar issues.

#### Run parameter settings

All run parameters were set according to their default settings, with SeqGapSizesChromBreak set to 0 by default. Notably, when applying these default parameters to species within Euphorbiaceae, the output resulted in an empty result. However, the study successfully produced results when using sample data parameters. Given the absence of a precise formula for SeqGapSizesChromBreak, its determination hinges on specific genome characteristics and the presence of multiple sequence duplications. Drawing from existing studies, we propose the following approaches to calculate this parameter: (Distance between metabolic genes) Calculated as genome size divided by the total number of metabolic genes; (Impact of multiple sequence duplication) Expressed as the impact factor, derived from the size of duplicate gene families divided by the genome size; (Maximum number of nonmetabolized genes allowed) Computed as the product of the distance, impact factor, and the size of the nonmetabolized gene family. These methods are intended to refine parameter selection, tailored to the genome's specific attributes and duplication patterns.

#### PlantClusterFinder software gene cluster prediction availability

Tools such as PlantClusterFinder and plantiSMASH are continuously improving in predicting these clusters involved in the same metabolic pathways, yet there remains a significant gap in understanding such complex genomes as Euphorbiaceae. Although PlantClusterFinder demonstrates strong predictive capabilities in the context of Euphorbiaceae species, it also yields numerous false-positive results. Therefore, the key focus of our study lies in establishing reasonable and feasible filtering criteria to confidently identify secondary metabolism gene clusters. The coexpression and colocalization principles in gene cluster prediction enhance true-positive rates, stringent filtering conditions often exclude potentially valuable data, resulting in a loss of false-negative results. Our research program integrates metabolic pathway databases, transcriptome data, and prediction tools, yet remains insufficient to fully exploit the potential of gene clusters due to the current limitations in genome sequencing quality for Euphorbiaceae.

### Universal applicability of the prediction framework to species

A prediction model specifically targeting Euphorbiaceae plants has been designed. The core strength of this framework lies in its targeted integration of the genomic features of Euphorbiaceae species and the synthesis pathways of terpenes. Our constructed framework has a clear structure and distinct steps, enabling efficient identification of terpene gene clusters in Euphorbiaceae. We state that although this framework is customized for Euphorbiaceae species, the fundamental methods and concepts (such as gene cluster identification and metabolic pathway inference) can be applied to a certain extent in other species. When applied to other species, certain adjustments and optimizations may be necessary based on the specific genomic characteristics to enhance the prediction accuracy.

### The assessment on the predictive process framework

#### Metabolic network reconstruction pipeline

The pipeline integrates an enzyme annotation process E2P2 [4] and pathway prediction software Pathway Tools [21], and a pathway prediction validation process called SAVI, which has doubled the number of enzymes in the gold standard protein sequences (RPSD v3.1). E2P2 v3.0 introduces Enzyme Function (EF) classes. This expansion brings the total EF classes to 11 902, a >5-fold increase from the previous version. The performance of E2P2 was evaluated using enzyme and non-enzyme sequences from RPSD with a 5-fold cross-validation. The enzyme annotation precision of 78.2%, recall of 69.3%, and an F1 score of 73.5%. This improvement optimizes the trade-off between precision and recall, outperforming individual methods like BLAST [22] and PRIAM [23]. Pipeline associates the predicted enzymes with reference reactions and pathways in MetaCyc (Caspi *et al.*, 2012) [24] using PathoLogic [21].

#### Prediction of plant metabolic gene clusters

PlantClusterFinder is a tool designed to identify metabolic gene clusters using an iterative approach. It ensures high-quality data by excluding genomes that do not meet specific assembly criteria. For example, the genomes of switchgrass and two wheat progenitors were excluded because >50% of their genomes were assembled into scaffolds with <50 genes per scaffold. Similarly, the barley genome was excluded due to its shotgun assembly, which contained excessive gaps, with every third intergenic region being incomplete on average. PlantClusterFinder also utilizes the sequence of genes located on a scaffold or chromosome, and sequencing gaps within the genome must be accounted for to avoid predictions that span uncalculated gaps. The software then generates sequencing gap location files based on the genome assembly [25–27], where sequencing gaps are represented as DNA fragments encoded by the letter ‘N’. This selective approach ensures that only genomes with sufficient assembly quality are included, enhancing the reliability and accuracy of the identified gene clusters.

#### Inferring high-confidence gene clusters using coexpression

The framework incorporates coexpression gene analysis in the process of identifying and validating gene clusters. By calculating Pearson correlation coefficient [28] between genes, the framework was able to identify coexpressed gene pairs associated with metabolic pathways and select gene pairs with high correlation through statistical analysis. Specifically, the framework uses the correlation of the top 99th percentile as a screening threshold, thus ensuring the accuracy and statistical significance of the gene clusters.

### Euphorbiaceae horticultural plants and terpene gene clusters

The Euphorbiaceae family comprises a diverse array of plants celebrated for their horticultural and medicinal significance. One notable example is *E. peplus*, a drought-tolerant shrub adorned with succulent gray-green leaves that not only enhance aesthetics but also reduce water evaporation, making it well suited for light environments. Its spherical or conical inflorescences boast striking yellow, green, or purple hues, further adding to its allure. Another prominent member is *R. communis*, recognized for its towering herbaceous stature and vibrant foliage, making it a prized ornamental in gardens. Beyond its decorative appeal, castor is valued for its medicinal properties; ricin found in its seeds exhibits antitumor potential, while castor oil serves as a feedstock for biodiesel, underscoring its industrial utility.

Furthermore, the terpenoid gene cluster within Euphorbiaceae has captivated scientific interest. Terpenoids play pivotal roles in plant physiology, offering antioxidant, antibacterial, and antifungal properties crucial for adapting to environmental challenges. Research into terpene synthesis genes in Euphorbiaceae promises insights into molecular mechanisms governing drought tolerance and stress resilience, paving the way for developing resilient and economically viable horticultural and agricultural varieties. Continued research, coupled with advances in gene editing technologies, is poised to unlock their full potential across diverse fields, ensuring their continued relevance and impact.

## Conclusion

To construct a comprehensive and confident pipeline to identify terpene gene clusters in Euphorbiaceae, we integrated seven genome and 71 transcriptome data, of which the genome and 42 transcriptome data from *V. montana* were firstly offered. The usage and debugging of prediction error for the tools including E2P2 v3.0, Pathway Tools, and PlantClusterFinder, were well presented. A total of 1824 candidates and 16 high-confidence gene clusters were finally determined. Among them, the specific casbene and casbene-derived diterpenoid gene cluster, responsible for the biosynthesis of casbene, neocembrene, ingenanes, and jatrophanes in Euphorbiaceae, were analyzed in detail on the genome structure, the coexpression rules, and the evolutionary features. These findings extended the applicability of gene cluster prediction to nonmodel species, and presented a significant step toward understanding the genetic basis of terpene biosynthesis in Euphorbiaceae species. Moreover, the interdisciplinary approach provides novel insights into gene cluster predictions and helps unravel the complexities of plant secondary metabolic pathways.

## Materials and methods

### Genome data from Euphorbiaceae species in website

Genome and protein data of Euphorbiaceae species were obtained from the NCBI (National Center for Biotechnology Information) and Phytozome databases (https://phytozome-next.jgi.doe.gov/). These data include *E. peplus* (GCA_028411795.1), *R. communis* (GCF_019578655.1), *M. esculenta* (GCF_001659605.2), *H. brasiliensis* (GCF_030052815.1), and *J. curcas* (GCF_014843425.1). Additionally, we acquired protein files (in protein format) and annotation files in gff3 format. To enhance our analysis of the evolutionary relationships within the *R. communis* casbene gene cluster, we augmented our dataset by downloading genomic data of base group species such as *Ananas comosus*, *Vitis vinifera*, and *Triticum aestivum*. This additional data will facilitate a comprehensive study of evolutionary patterns across species.

### Transcriptome data in website

For some, transcriptome data used in this study were primarily sourced from NCBI. Specifically, we utilized data for *R. communis* transcriptome (SRR17218210-SRR17218218, SRR17218221-SRR17218223, SRR21177948-SRR21177950, SRR21177960, SRR21177961) and *E. peplus* transcriptome (SRR21854228-SRR21854245).

### *Vernicia montana* genome data

We used a number of metrics to comprehensively assess the quality and integrity of the assembly, including the Benchmarking Universal Single-Copy Orthologs (BUSCO) assessment, LTR Assembly Index (LAI) assessment, and Merqury assessment. According to the BUSCO evaluation results, we obtained the following results for the Hap1 and Hap2 haplotype assembly of the *V. montana*: Hap1: Complete (C) 1589 [Single-copy (S): 1562, Duplicated (D): 271, Fragmented (F): 10, Missing (M): 15], Hap2: Complete (C) 1591 [Single-copy (S): 1570, Duplicated (D): 21, Fragmented (F): 9, Missing (M): 14] (Table 4). This result shows that the assembly quality of both haplotypes is high, especially the high number of intact genes and the low number of missing genes.

**Table 4:** Evaluation parameter of *V. montana* genome.

	Hap1		Hap2	
	**Evalue**	**Percentage**	**Evalue**	**Percent**age
Complete BUSCOs	1589	98.1	1591	98.3
Complete and single-copy BUSCOs	1562	96.4	1570	96.6
Complete and duplicated BUSCOs	27	1.7	21	1.7
Fragmented BUSCOs	10	0.6	9	0.6
Missing BUSCOs	15	1.3	14	1.1
Total BUSCOs	1614	100	1614	100
LAI (LTR Assembly Index)	16.43		16.36	
Merqury (QV)	62.6553		62.627	

LAI is an important index used to evaluate the assembly integrity of long-terminal repeating retrotransposon (LLTR-RTS) class repeat sequences in the genome. The LAI values of Hap1 and Hap2 of the genome were 16.43 and 16.36, respectively (Fig. S6), indicating that the assembly of the two haplotypes did a fairly good job in processing these repeats, with no significant repeat omissions in the assembly.

Merqury is a comparison-based evaluation method that calculates quality value (QV) by comparing third-generation sequenced HiFi reads back to assembly results. The mass value of Hap1 was 62.6553, and that of Hap2 was 62.627, indicating that the genome assembly of *V. montana* performed very well in terms of accuracy and consistency (Fig. S6).

In summary, based on the comprehensive analysis of the above three evaluation indicators, the assembly results of the genome of *V. montana* have excellent performance in integrity, accuracy, and ability to process repeated sequences (Please refer to the detailed evaluation data: Fig. S6). The genome data are available on NCBI under project number: PRJNA1147434.

### Transcriptome data and preprocessing

A total of 42 samples from 14 tissues, including axial root, lateral root, apex, young leaf, mature leaf, leaf bud, gland, stem, fruitlet, fruit, kernel, and fruit peel, were collected for transcriptome analysis. Full-length transcriptome sequencing was performed using the PacBio platform, with paired sequencing libraries constructed by PacBio. The sequencing data underwent quality control before being analyzed bioinformatically. The data is available on NCBI under project number: PRJNA1146716.

For the high-quality screening phase, the transcriptome data underwent rigorous quality control to minimize noise. Using Trimmomatic [29], we removed adapter sequences and low-quality fragments from the sequencing data (Table 5). Subsequently, the cleaned data was aligned to the reference genome using HISAT [30], followed by read counting with Subread [31] to quantify reads per transcript. Expression levels were normalized to Fragments Per Kilobase of Transcript per Million mapped reads (FPKM), ensuring comparability across samples based on transcript–gene correspondence. With these steps completed, the transcriptome data is now prepared for further analysis.

**Table 5:** Trimmatic operating parameters and description.

Command	Parameter	Description
phread	33	Fasta file default 33
ILLUMINACLIP	2:30:10	Shear parameter
LEADING/TRAILING	5	Below the threshold, respectively shear
SLIDINGWINDOW	5:20	Average mass <5, cut
MINLEN	36	Reads below length 36 are deleted
AVGQUAL	20	Reads <20 are discarded

### Data preprocessing

**First, annotation files** are required. The annotation file (glof.txt) needs to retain specific columns: gene name, start position, chromosome, and information on the forward and reverse strands. Additionally, for protein–gene correspondence, ensure the file includes three columns: gene name, transcript ID (optional), and protein ID.

**Next for Enzyme annotations**. Protein annotation for the species is performed using the E2P2 software pipeline module (Fig. S4a). This process generates four output files: e2p2v4, e2p2v4.long, e2p2v4.orxn.pf, and e2p2v4.pf (Fig. S4b, c). Protein data for seven representative species of Euphorbiaceae were annotated using the E2P2 pipeline. Subsequently, a metabolic pathway database was constructed using Pathway Tools software, renowned for its comprehensive suite of integrated functionalities ranging from genomic analysis to metabolic modeling and high-throughput data analysis.

**Subsequently for PGDB data**. Next, the 'orxn.pf' file undergoes further processing using Pathway Tools software to establish a metabolic pathway database. This involves navigating through the Pathologic tool: 'Tools' → 'Pathologic' → 'Database' → 'Create' → 'new'. Finally, export the constructed database from the Home directory by selecting: File → Export → Entire DB attribute-value and BioPAX files to finalize the PGDB data construction (Fig. S5). Pathway Tools enables the generation of PGDBs directly from genome data in GenBank or GFF format. This capability facilitates in-depth analysis and visualization of biological pathways and genetic interactions. Currently, over 14 500 PGDBs are accessible via the BioCyc database collection curated by SRI International. These databases span a wide range of organisms and biological processes, bolstering research in fields like bioinformatics, systems biology, and metabolic engineering [21]. Furthermore, Pathway Tools allows users to develop custom PGDBs tailored to their specific research requirements. In our study, following the prediction process we established, we initially tested it using test data. The program performed flawlessly, yielding results consistent with those obtained by the software developers during testing. This validation underscores the practical viability of the prediction environment we constructed.

**Finally, for software operation**, it is recommended to execute it using the full path to Matlab-2023b by running the ‘run_PlantClusterFinder.sh’ program with various input data parameters as specified of the PlantClusterFinder1.3 run parameters and instructions (Table 6). Including the Verbose 2 parameter in the command is highly advisable, as it provides detailed progress updates crucial for monitoring the program's execution status. The prediction process typically takes ~3 to 8 h to complete, depending on factors such as the genome size of the species and the computational resources available on the server. This timeframe may vary due to specific conditions such as server load and genome complexity. By following these guidelines and ensuring the inclusion of the ‘Verbose 2’ parameter, users can effectively monitor the execution of PlantClusterFinder1.3 and manage the prediction process efficiently. This approach facilitates accurate results and optimal use of computational resources.

**Table 6:** PlantClusterFinder1.3 operating parameters and description.

**Parameters**	**Value**	**Explanation**
-pgdb	Species_pgdb	Metabolic databases
-psf	Species_protein.fa	Protein files
-gtpf.txt	Gene_transcript_protein	Gene–Protein correspondence
-glof.txt	Gff3	Annotation information
-dnaf	Species.fa	Genome files
-gout	Species_Gene	Gene enzyme annotation
-cout	Species_Clusters	Gene cluster results

### High-confidence terpenoid secondary metabolism gene cluster screening

The screening criteria for plant secondary metabolic gene clusters included: (1) each gene cluster must contain at least three metabolic genes. (2) At least two different MetaCyc response identifiers are required within the gene cluster. (3) All genes in the gene cluster must be located in a continuous region on the same chromosome. (4) The inclusion of only locally repeated metabolic genes is not allowed (Schlapfer *et al.*, 2017). In addition, we also refer to the structural characteristics of plant secondary metabolite gene clusters. The length of gene clusters is usually distributed in the range of 35–500 kb, among which the 33–284 kb range is more common, and the length of most gene clusters is close to 100 kb. The number of metabolic genes within gene clusters is generally between 4 and 18 (Nutzmann H W *et al.*, 2016). To effectively screen for high-quality terpene secondary metabolism gene clusters, we have established rigorous criteria:

#### Copathway criteria

Gene clusters must include at least one pair of genes involved in the same metabolic pathway. These genes within the pathway should catalyze distinct metabolic reactions. Verification of pathway involvement and reaction specificity is based on annotation data such as MetaCyc PWY IDs for pathways and RXN IDs for reactions.

#### Coexpression analysis

Coexpression is pivotal for indicating functional coordination within gene clusters. We employ Weighted Gene Coexpression Network Analysis (WGCNA) [32] to identify genes grouped within the same expression module. Validation involves visualizing coexpression patterns using the Pearson Correlation Coefficient, supported by studies like Usadel *et al.* [28] and Wisecaver *et al.* [33], which demonstrate its reliability in predicting gene functions in metabolic pathways.

#### Genomic arrangement and structural confirmation

TPS (Terpenoid Synthesis Genes) and CYP (Cytochrome P450) genes commonly colocalize in plant genomes, underscoring their functional linkage in terpenoid synthesis. Structural domain analysis via InterProScan V5 [34] confirms gene identities: TPS genes are identified by pfam IDs PF01397 and PF03936, while CYP genes are identified by pfam ID PF00067.

#### Integration of predictions

To enhance annotation accuracy, we integrate predictions from gene cluster analysis with structural domain confirmation. Priority is given to gene clusters where TPS and CYP genes co-occur, indicating potential for efficient terpenoid synthesis. By adhering to these criteria, we ensure robust screening and prioritization of gene clusters involved in terpene secondary metabolism, facilitating comprehensive understanding and utilization of these pathways.

### Evolutionary analysis of gene clusters

To explore the evolutionary relationships and history of species, we conducted a colinearity analysis of high-quality metabolic synthesis gene clusters across multiple species. This analysis was performed using the MCscanX tool, integrated into TBtools as described by Chen *et al.* [35]. Our objective was to investigate the collinearity patterns of these gene clusters, which allowed us to gain insights into their evolutionary associations and changes over time.

Specifically, we utilized the angiosperm phylogenetic tree framework, incorporating the APG IV system for flowering plants. By leveraging this framework, we aimed to compare genome-wide collinearity among several species. This approach enabled us to unravel the evolutionary dynamics and implications of these metabolic synthesis gene clusters.

Through this comprehensive analysis, we aimed to contribute to a deeper understanding of how these gene clusters have evolved across different species, shedding light on their functional and evolutionary significance in metabolic pathways within angiosperms.

## Supplementary Material

Web_Material_uhaf097

## Data Availability

All data are presented inside the manuscript and its supplementary data. The *V. montana* reference genome data is available on NCBI under project number: PRJNA1147434. The *V. montana* transcriptome data is available on NCBI under project number: PRJNA1146716.

## References

[1] Wang HB, Wang XY, Liu LP. et al. Tigliane diterpenoids from the Euphorbiaceae and Thymelaeaceae families. Chem Rev. 2015;115:2975–3011 25906056 10.1021/cr200397n

[2] Jones CG, Martynowycz MW, Hattne J. et al. The CryoEM method MicroED as a powerful tool for small molecule structure determination. ACS Central Science. 2018;4:1587–92 30555912 10.1021/acscentsci.8b00760 PMC6276044

[3] Siller G, Rosen R, Freeman M. et al. PEP005 (ingenol mebutate) gel for the topical treatment of superficial basal cell carcinoma: results of a randomized phase IIa trial. Australas J Dermatol. 2010;51:99–105 20546215 10.1111/j.1440-0960.2010.00626.x

[4] Chae L, Kim T, Nilo-Poyanco R. et al. Genomic signatures of specialized metabolism in plants. Science. 2014;344:510–3 24786077 10.1126/science.1252076

[5] Medema MH, Kottmann R, Yilmaz P. et al. Minimum information about a biosynthetic gene cluster. Nat Chem Biol. 2015;11:625–31 26284661 10.1038/nchembio.1890 PMC5714517

[6] Schläpfer P, Zhang P, Wang C. et al. Genome-wide prediction of metabolic enzymes, pathways, and gene clusters in plants. Plant Physiol. 2017;173:2041–59 28228535 10.1104/pp.16.01942 PMC5373064

[7] Kautsar SA, Suarez Duran HG, Blin K. et al. plantiSMASH: automated identification, annotation and expression analysis of plant biosynthetic gene clusters. Nucleic Acids Res. 2017;45:W55–63 28453650 10.1093/nar/gkx305 PMC5570173

[8] Töpfer N, Fuchs LM, Aharoni A. The PhytoClust tool for metabolic gene clusters discovery in plant genomes. Nucleic Acids Res. 2017;45:7049–63 28486689 10.1093/nar/gkx404 PMC5499548

[9] Luo J . Metabolite-based genome-wide association studies in plants. Curr Opin Plant Biol. 2015;24:31–8 25637954 10.1016/j.pbi.2015.01.006

[10] Frey M, Schullehner K, Dick R. et al. Benzoxazinoid biosynthesis, a model for evolution of secondary metabolic pathways in plants. Phytochemistry. 2009;70:1645–51 19577780 10.1016/j.phytochem.2009.05.012

[11] Field B, Osbourn AE. Metabolic diversification—independent assembly of operon-like gene clusters in different plants. Science. 2008;320:543–7 18356490 10.1126/science.1154990

[12] Wurdack KJ, Hoffmann P, Chase MW. Molecular phylogenetic analysis of uniovulate Euphorbiaceae (Euphorbiaceae sensu stricto) using plastid rbcL and trnL-F DNA sequences. Am J Bot. 2005;92:1397–420 21646159 10.3732/ajb.92.8.1397

[13] Boycheva S, Daviet L, Wolfender JL. et al. The rise of operon-like gene clusters in plants. Trends Plant Sci. 2014;19:447–59 24582794 10.1016/j.tplants.2014.01.013

[14] Czechowski T, Forestier E, Swamidatta SH. et al. Gene discovery and virus-induced gene silencing reveal branched pathways to major classes of bioactive diterpenoids in Euphorbia peplus. Proc Natl Acad Sci. 2022;119:e2203890119 35584121 10.1073/pnas.2203890119 PMC9173813

[15] King AJ, Brown GD, Gilday AD. et al. Production of bioactive diterpenoids in the Euphorbiaceae depends on evolutionarily conserved gene clusters. Plant Cell. 2014;26:3286–98 25172144 10.1105/tpc.114.129668 PMC4371829

[16] Boutanaev AM, Moses T, Zi J. et al. Investigation of terpene diversification across multiple sequenced plant genomes. Proc Natl Acad Sci. 2015;112:E81–8 25502595 10.1073/pnas.1419547112 PMC4291660

[17] Tokuoka T, Tobe H. Phylogenetic analyses of Malpighiales using plastid and nuclear DNA sequences, with particular reference to the embryology of Euphorbiaceae sens. str. J Plant Res. 2006;119:599–616 16937025 10.1007/s10265-006-0025-4

[18] Prochnik S, Marri PR, Desany B. et al. The cassava genome: current progress, future directions. Trop Plant Biol. 2012;5:88–94 22523606 10.1007/s12042-011-9088-z PMC3322327

[19] Strommer J . The plant ADH gene family. Plant J. 2011;66:128–42 21443628 10.1111/j.1365-313X.2010.04458.x

[20] Zhao Y, Chen Y, Gao M. et al. Alcohol dehydrogenases regulated by a MYB44 transcription factor underlie Lauraceae citral biosynthesis. Plant Physiol. 2024;194:1674–91 37831423 10.1093/plphys/kiad553

[21] Karp PD, Midford PE, Billington R. et al. Pathway tools version 23.0 update: software for pathway/genome informatics and systems biology. Brief Bioinform. 2021;22:109–26 31813964 10.1093/bib/bbz104 PMC8453236

[22] Altschul SF, Gish W, Miller W. et al. Basic local alignment search tool. J Mol Biol. 1990;215:403–10 2231712 10.1016/S0022-2836(05)80360-2

[23] Claudel-Renard C, Chevalet C, Faraut T. et al. Enzyme-specific profiles for genome annotation: PRIAM. Nucleic Acids Res. 2003;31:6633–9 14602924 10.1093/nar/gkg847 PMC275543

[24] Caspi R, Altman T, Billington R. et al. The MetaCyc database of metabolic pathways and enzymes and the BioCyc collection of pathway/genome databases. Nucleic Acids Res. 2014;42:D459–71 24225315 10.1093/nar/gkt1103 PMC3964957

[25] Goodstein DM, Shu S, Howson R. et al. Phytozome: a comparative platform for green plant genomics. Nucleic Acids Res. 2012;40:D1178–86 22110026 10.1093/nar/gkr944 PMC3245001

[26] Harper L, Gardiner J, Andorf C. et al. MaizeGDB: the maize genetics and genomics database. Plant bioinformatics: Methods and protocols. 2016;1374:187–202 10.1007/978-1-4939-3167-5_9 26519406

[27] Kersey PJ, Allen JE, Armean I. et al. Ensembl genomes 2016: more genomes, more complexity. Nucleic Acids Res. 2016;44:D574–80 26578574 10.1093/nar/gkv1209 PMC4702859

[28] Usadel B, Obayashi T, Mutwil M. et al. Co-expression tools for plant biology: opportunities for hypothesis generation and caveats. Plant Cell Environ. 2009;32:1633–51 19712066 10.1111/j.1365-3040.2009.02040.x

[29] Bolger AM, Lohse M, Usadel B. Trimmomatic: a flexible trimmer for Illumina sequence data. Bioinformatics. 2014;30:2114–20 24695404 10.1093/bioinformatics/btu170 PMC4103590

[30] Pertea M, Kim D, Pertea GM. et al. Transcript-level expression analysis of RNA-seq experiments with HISAT, StringTie and Ballgown. Nat Protoc. 2016;11:1650–67 27560171 10.1038/nprot.2016.095 PMC5032908

[31] Liao Y, Smyth GK, Shi W. The R package Rsubread is easier, faster, cheaper and better for alignment and quantification of RNA sequencing reads. Nucleic Acids Res. 2019;47:e47–7 30783653 10.1093/nar/gkz114 PMC6486549

[32] Langfelder P, Horvath S. WGCNA: an R package for weighted correlation network analysis. BMC bioinformatics. 2008;9:1–13 19114008 10.1186/1471-2105-9-559 PMC2631488

[33] Wisecaver JH, Wisecaver JH, Borowsky AT. et al. A global coexpression network approach for connecting genes to specialized metabolic pathways in plants. Plant Cell. 2017;29:944–59 28408660 10.1105/tpc.17.00009 PMC5466033

[34] Zdobnov EM, Apweiler R. InterProScan–an integration platform for the signature-recognition methods in InterPro. Bioinformatics. 2001;17:847–8 11590104 10.1093/bioinformatics/17.9.847

[35] Chen C, Wu Y, Li J. et al. TBtools-II: a “one for all, all for one” bioinformatics platform for biological big-data mining. Mol Plant. 2023;16:1733–42 37740491 10.1016/j.molp.2023.09.010

