# Supplementary material for: Genome-wide terpene gene clusters analysis in Euphorbiaceae
Source: Hortic Res. 2025 May 23;12(7):uhaf097. doi: 10.1093/hr/uhaf097 (PMC12117367; doi:10.1093/hr/uhaf097)
Supplement: Web_Material_uhaf097 [file web_material_uhaf097.zip › Supplement Fig.pdf]

Fig S1 ： Physical length of gene clusters vs. number of genes

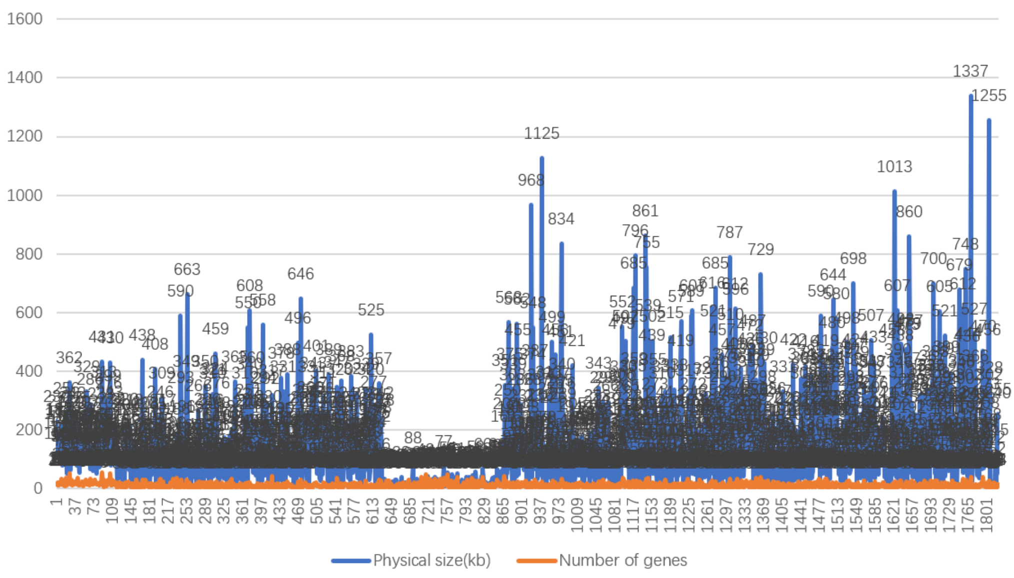

Fig S2 ： Statistics on the number of signalling enzymes in Euphorbiaceae species

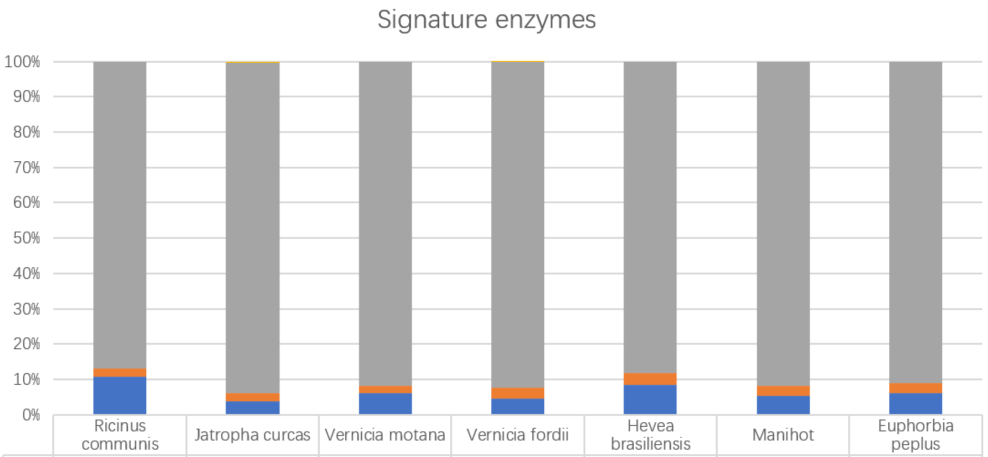

	Ricinus communis	Jatropha curcas	Vernicia motana	Vernicia fordii	Hevea brasiliensis	Manihot	Euphorbia peplus
PKS	0	4	0	1	0	0	0
ASE	584	889	495	533	815	701	502
PSE	15	21	11	18	30	23	16
TPS	73	37	33	27	78	40	34

TPS PSE ASE PKS

Fig S3： *V. fordii* clusters Pearson analysis

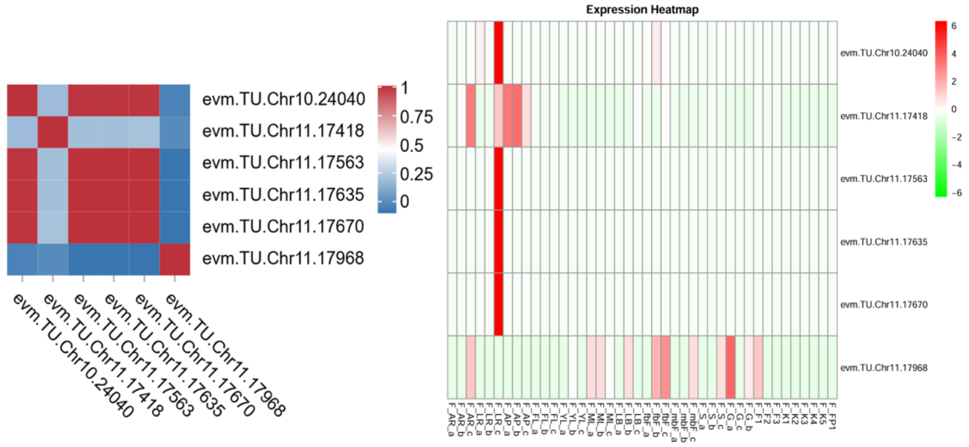

Correspondence of each gene in the casbene gene cluster of *V. fordii* and *R. communis*

evm.TU.Chr10.24940—Monoterpene synthase
evm.TU.Chr11.17418—Monoterpene synthase
evm.TU.Chr11.17563—ADH
evm.TU.Chr11.17635—Casbene synthase
evm.TU.Chr11.17670——CYP726A18
evm.TU.Chr11.17968——Casbene synthase

Fig S4 : Protein EC annotation results output by E2P2 (Take R. communis as an example) (a)Protease annotation details(b) E2P2 output file (c)E2P2 operation process

a

```
ID         WCJ17614. 1
NAME       WCJ17614. 1
PRODUCT-TYPE    P
METACYC GUANYLCYC-RXN
//
ID         WCJ17615. 1
NAME       WCJ17615. 1
PRODUCT-TYPE    P
METACYC 1. 1. 1. 210-RXN
METACYC ALLYL-ALCOHOL-DEHYDROGENASE-RXN
METACYC ENZRXN-201-RXN
METACYC RXN-12078
METACYC RXN-12297
METACYC RXN-12299
METACYC RXN0-4281
//
ID         WCJ17622. 1
NAME       WCJ17622. 1
PRODUCT-TYPE    P
METACYC 1. 14. 13. 93-RXN
//
ID         WCJ17624. 1
NAME       WCJ17624. 1
PRODUCT-TYPE    P
METACYC RXN-9021
METACYC RXN-9022
//
ID         WCJ17641. 1
NAME       WCJ17641. 1
PRODUCT-TYPE    P
METACYC CARBOXYLESTERASE-RXN
METACYC RXN-3284
METACYC RXN-3303
```

b

```
R. communis_protein. faa. e2p2v4
R. communis_protein. faa. e2p2v4. long
R. communis_protein. faa. e2p2v4. orxn. pf
R. communis_protein. faa. e2p2v4. pf
```

c

```
MainProcess Running level-0 classification processes.
MainProcess Compiling predictions.
MainProcess Computing ensemble predictions.
MainProcess Preparing results files.
MainProcess Operation complete.
MainProcess Main results are in the file: R. communis_protein. faa. e2p2v4
MainProcess Detailed results   : R. communis_protein. faa. e2p2v4. long
MainProcess To build PGDB, use:R. communis_protein. faa. e2p2v4. orxn. pf
```

Fig S5 :Pathway Tools Version 23.0 software interface

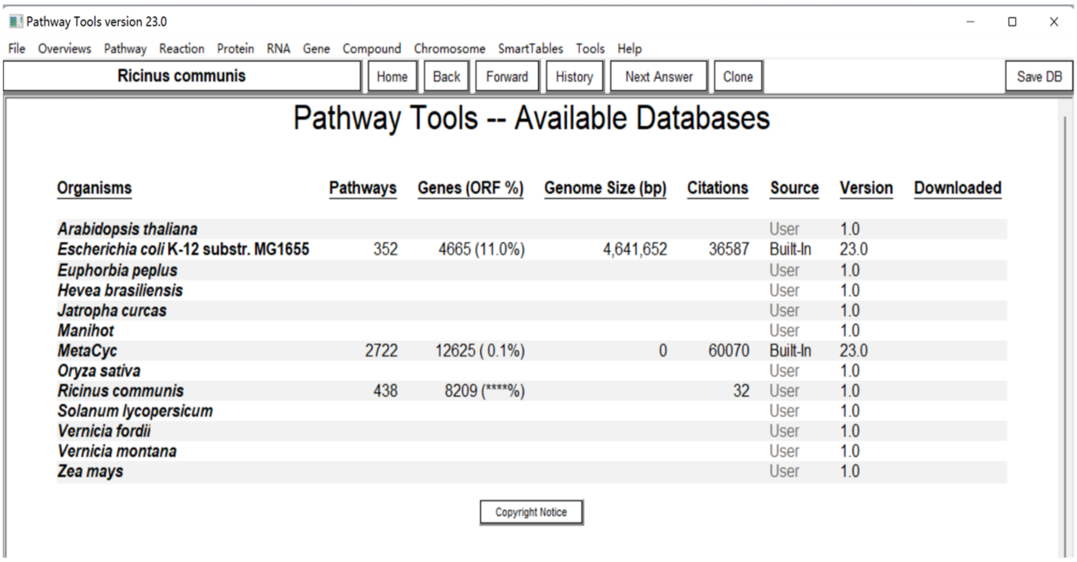

Organisms	Pathways	Genes (ORF %)	Genome Size (bp)	Citations	Source	Version	Downloaded
Arabidopsis thaliana					User	1.0	
Escherichia coli K-12 substr. MG1655	352	4665 (11.0%)	4,641,652	36587	Built-In	23.0	
Euphorbia peplus					User	1.0	
Hevea brasiliensis					User	1.0	
Jatropha curcas					User	1.0	
Manihot					User	1.0	
MetaCyc	2722	12625 (0.1%)	0	60070	Built-In	23.0	
Oryza sativa					User	1.0	
Ricinus communis	438	8209 (****%)		32	User	1.0	
Solanum lycopersicum					User	1.0	
Vernicia fordii					User	1.0	
Vernicia montana					User	1.0	
Zea mays					User	1.0	
